# Acute Effects of Vibration Foam Rolling with Light and Moderate Pressure on Blood Pressure and Senior Fitness Test in Older Women

**DOI:** 10.3390/ijerph182111186

**Published:** 2021-10-25

**Authors:** Wen-Chieh Yang, Che-Hsiu Chen, Lee-Ping Chu, Chih-Hui Chiu, Chin-Hsien Hsu, Kai-Wei Yu, Xin Ye

**Affiliations:** 1 Department of Physical Therapy, Hung Kuang University, Taichung 433304, Taiwan; wcyang@sunrise.hk.edu.tw; 2 Department of Sport Performance, National Taiwan University of Sport, Taichung 404401, Taiwan; jakic1114@ntus.edu.tw; 3 Department of Orthopedics, China Medical University Hospital, Taichung 404333, Taiwan; chu.leeping@gmail.com; 4 Department of Exercise Health Science, National Taiwan University of Sport, Taichung 404401, Taiwan; chiuch@ntus.edu.tw; 5 Department of Leisure Industry Management, National Chin-Yi University of Technology, Taichung 411030, Taiwan; 6 Department of Leisure Industry Management and Institute of Project Management, National Chin-Yi University of Technology, Taichung 411030, Taiwan; kaiwei40150@gmail.com; 7 Department of Rehabilitation Sciences, University of Hartford, West Hartford, CT 06117, USA

**Keywords:** warm up, stretching, arterial pressure, flexibility, strength

## Abstract

Vibration foam rolling (VR) can improve flexibility and sports performance. However, blood pressure (BP), heart rate (HR) and senior fitness test (SFT) responses induced by an acute VR session in older women are currently unknown. Fifteen healthy women (72.90 ± 4.32 years) completed three separated randomly sequenced experimental visits. During each visit, they started with a warm-up protocol (general warm up (GW): walking + static stretching (SS), SS + VR with light pressure (VRL), or SS + VR with moderate pressure (VRM)), and completed BP, HR, SFT measurements. The systolic BP increased significantly after all three warm up protocols (*p* < 0.05). Both VRL and VRM protocols induced statistically significant improvements (effect size range: 0.3–1.04, *p* < 0.05) in the senior fitness test (back scratch, 30 s chair stand, 30 s arm curl, and 8 foot up and go), as compared to the GW. In addition, the VRM showed greater improvement for the 2 min step test when comparing with the VRL. Therefore, including VR in a warm-up protocol can result in superior SFT performance enhancement than the GW does in healthy older women.

## 1. Introduction

Foam rollers (FR) are a common type of warm-up and relaxation tool. Users can press their limbs against the surface of a roller, apply weight, and roll back and forth to apply pressure to the target muscles. FRs are believed to provide the following benefits: increased joint range of motion (ROM), reduced pain at the trigger point, and alleviation of delayed-onset muscle soreness [1,2]. Therefore, FRs are often used for increasing joint ROM, warm-ups before exercise, and recovery after exercise.

The benefits of FRs in terms of physiological mechanisms include the adaptation of soft tissues after compression, the decrease in tissue viscoelasticity and lactic acid accumulation after an increase in local blood circulation, and the decrease in local and systemic muscle tension caused by the regulation of reflex arcs and sympathetic nerves to inhibit muscle tissue after pressure sensation input [3].Regarding mobility, Wilke et al. conducted a meta-analysis and discovered that the intervention of FRs can immediately increase mobility equivalent to that of static stretching (SS); furthermore, the increase in mobility among women is greater than that among men [3].The benefits of FRs on muscle performance are not influenced by the rolling direction (vertical or parallel to the direction of muscle fibers) [4], implying that the mechanism may be related to changes in neuromuscular regulation rather than to changes in the soft tissue structure. As for balance functions, Halperin et al. revealed that the intervention of FRs does not significantly improve balance performance. The possible reason is that balance performance is simultaneously associated with multiple factors, including mobility, muscle strength, and physical coordination [5]. Interestingly, Okamoto et al. indicated that FR interventions in the lower limbs significantly reduce brachial–ankle pulse wave velocity and significantly increase plasma nitric oxide concentration. These changes suggest that FRs not only benefit soft tissues (e.g., muscles and fasciae) but also reduce arterial stiffness and increase vascular endothelial function [6].

Vibration rolling (VR) has a built-in vibrating motor that generates vibration, which can stimulate proprioceptors such as the muscle spindles and Golgi tendon organs [7,8]. However, whether VR is more beneficial than nonvibrating rollers remains undetermined. Cheatham et al. discovered that compared with nonvibrating rollers, VR can more significantly increase quadricep ROM and the pressure pain threshold [9]. Nevertheless, Garcia-Gutiérrez et al. observed that an increase in dorsiflexion ROM attributable to VR is not significantly different from that attributable to non-VR; neither VR nor non-VR increase plantar flexion force [10].Moreover, Lee et al. discovered that both VR and SS significantly increase flexion ROM but non-VR does not. The effect of VR on improving balance and quadricep strength is significantly greater than that of SS, and the magnitude of the increase in the error of active knee joint reposition tests induced by VR intervention is significantly smaller than that induced by non-VR intervention [11]. Accordingly, the effectiveness of VR in improving ROM and muscle performance is equivalent to or slightly higher than that of non-VR and SS, and the degree of proprioceptive disturbance after VR intervention is smaller than that after non-VR intervention. 

Studies have mostly focused on the benefits of warm-up exercises [12,13,14,15] in healthy adults, and few studies have recruited older adults as participants. Ozsoy et al. examined the effects of the combined intervention of rollers and core exercise on nonspecific lower back pain in older adults. The results revealed that the effectiveness of the combined intervention in increasing spinal mobility was significantly higher than the sole intervention of core exercise. However, no significant differences in pain, disability, gait, or quality of life were observed between the combined and sole interventions [16]. When soft tissues are under stress, the performance of older adults in terms of compliance, peripheral circulatory function, and the reaction of the nervous system and the autonomic nervous system in the regulation of muscle tension when sensing stimulation input all differed from those of young adults [17,18]. Therefore, the research results claiming benefits of rollers for healthy young adults and athletes may not be applicable to older adults.

Numerous studies have indicated that higher stretching intensity (stretching more than 50% of the point of discomfort (POD)) significantly reduces muscle strength or exercise performance [19,20,21]. For example, Marchetti et al. discovered that among young men with resistance training experience, a moderate SS intensity (stretching to 50% of the POD) and a higher SS intensity (stretching to 80% of the POD) immediately and significantly increase passive hip flexion ROM; however, higher SS intensity significantly reduces peak force during maximal isometric leg curl exercises [19]. Similar research reveals that compared with light massages or vibratory stimulation (40 Hz) interventions that increased arousal and heart rate, moderate massages resulted in the greatest decrease in self-reported stress and heart rate [22]. However, few studies have explored the combined effects of using SS and VR with different intensities on older adults’ muscle function and blood pressure. 

Therefore, the main purpose of the study was to examine the acute effects of VR with different pressure intensities on systolic blood pressure, diastolic blood pressure, heart rate, and senior fitness test results in older female adults. We hypothesize that adding the higher-pressure intensity VR may improve the older women’s functional test performance without affecting the blood pressure responses.

## 2. Materials and Methods

### 2.1. Participants

Fifteen women (age: 72.90 ± 4.32 years, height: 153.29 ± 2.83 cm, body mass: 60.78 ± 4.94 kg, body mass index: 24.06 ± 3.96 kg/m^2^) were recruited to participate in this study. The inclusion criteria included the age range of 60–80 years of age. The exclusion criteria included the presence of any musculoskeletal injury or metabolic disease. Before any experimental testing, each participant signed an approved informed consent document. On all experimental visit days, participants were instructed not to consume any alcohol or caffeine. All the experimental procedures in this investigation were in accordance with the Declaration of Helsinki and approved by the China Medical University and Hospital Research Ethics Committee (CMUH109-REC3-107, 18 August 2020).

### 2.2. Experimental Procedures

**Figure 1:**
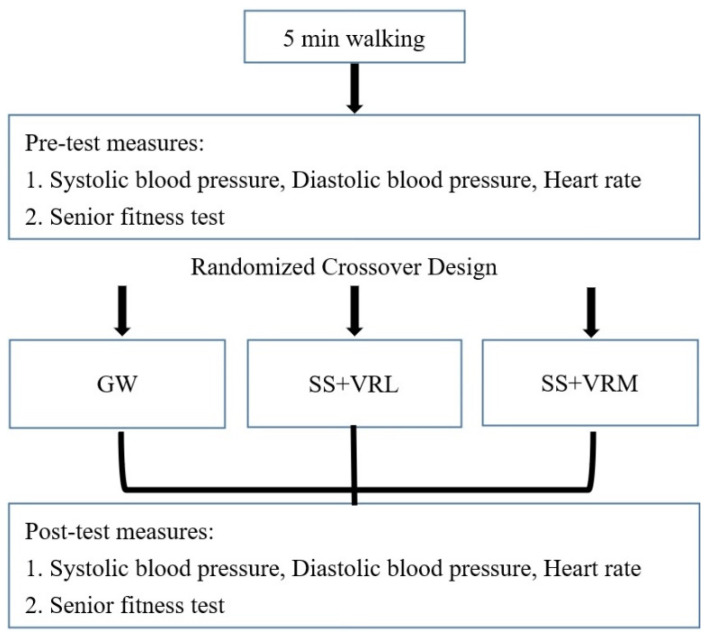
The experimental design of the study.

This study used a within-subject crossover design to investigate the acute effects of general warm up (GW), vibration foam rolling with light (VRL), and with moderate pressure (VRM) intensities on systolic blood pressure (SBP), diastolic blood pressure (DBP), heart rate (HR) and the senior fitness test (SFT). One week before the first experimental testing visit, all participants were familiarized with all the procedures. Next, participants completed three testing sessions on three separate days, each at least 7 days apart. Considering the majority of the tests conducted in the current study were low intensity exercises, a 7-day rest period would be enough for the recovery. In addition, extra effort was taken to ensure the tests were conducted at around the same time of day for each participant. Participants were refrained from vigorous physical activities 24 h before each test session. At the beginning of each session, participants performed 5 min walking (the intensity was instructed to maintain of 11 on a Borg 6–20 scale). Baseline testing (pre-test), which consisted of systolic and diastolic blood pressure, heart rate, back scratch, chair sit and reach, 30 s arm curl, 30 s chair stand, 2 min step, and 8 foot up and go testing, was conducted after the 5 min of walking. After completing the pretest measurements, participants performed one of the warm-up protocols (GW, VRL, or VRM) randomly selected for that session. Post-test measures were performed in the same order as the pre-test measures immediately after the intervention protocol. The flowchart of the study design is presented in Figure 1.

### 2.3. Measurements

#### 2.3.1. Blood Pressure and Heart Rate

Pre-test and post-test systolic blood pressure, diastolic blood pressure, and heart rate were measured using an automatic oscillometric device (Omron model HEM-8611, Omron Corporation, Taichung, Taiwan). 

#### 2.3.2. Senior Fitness Test (SFT)

In accordance with Rikli and Jones [23], the SFT included 6 motor tests: back scratch, chair sit and reach, 30 s chair stand, 30 s arm curl, 2 min step, and 8 foot up and go testing. These tests aim to determine physical parameters such as shoulder flexibility, lower-body flexibility, lower-body strength, upper-body strength, aerobic endurance, agility, and dynamic balance.

### 2.4. Warm-Up Protocols

#### 2.4.1. General Warm up (GW)

**Figure 2:**
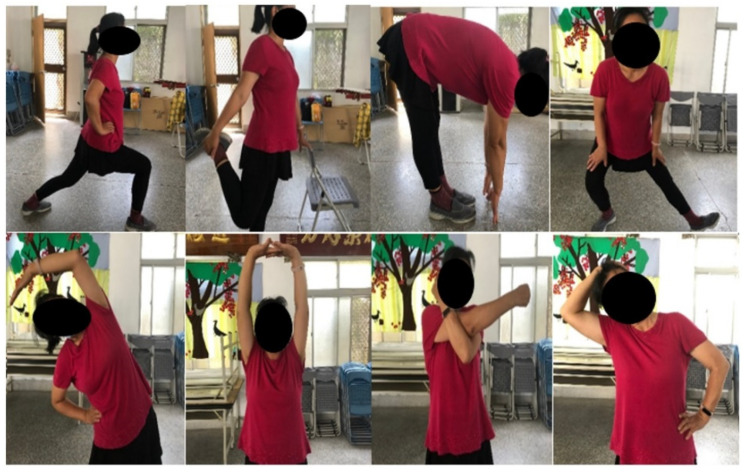
The demonstration of the static stretching protocol.

The GW consisted of a 16-min static stretching routine. Specifically, the self-administered static stretching involved the participants holding still at the end of the range of motion for 30 s for each body part (Figure 2). The participants performed 2 sets of eight stretching exercises rotationally, targeting calf, quadriceps, hamstrings, hip adductors, obliques, latissimus dorsi, upper-back muscles, posterior deltoid, and neck lateral flexors muscles. Additionally, the stretching exercises were performed to the threshold of mild discomfort, without feeling pain. A 30 s rest interval was provided for switching between the body parts.

#### 2.4.2. Vibration Rolling with Light Pressure Intensity (VRL)

**Figure 3:**
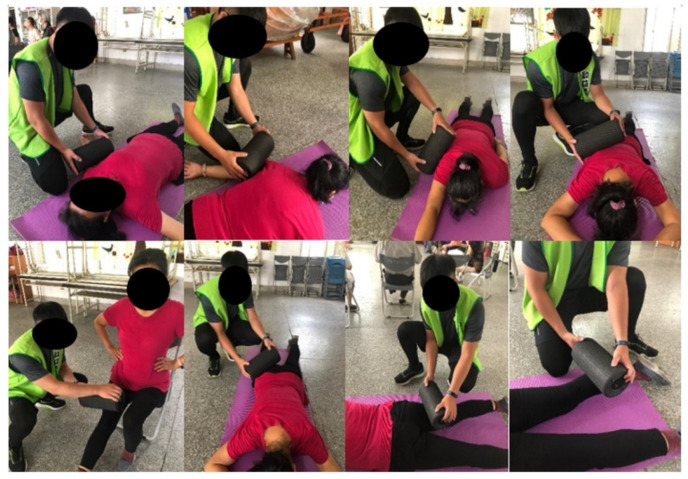
The demonstration of the vibration rolling protocols.

The VRL consisted of one set of self-administered static stretching exercises as mentioned above (8 min), followed by the passive vibration rolling (16 min). This study employed a commercial vibration foam roller (Vyper 2.0, Hyperice, Irvine, CA, USA) with the vibration frequency set at 48 Hz. The VR was administered passively by a research staff to roll the roller back and forth on the participants’ triceps brachii, biceps brachii, rotator cuff, latissimus dorsi, calf, quadriceps, gluteal, hamstrings muscles at both sides. The pressure pain intensity was instructed to maintain at 2–3 on a 10 mm visual analogue scale (VAS), based on each participant’s verbal feedback. Each set of rolling was performed for 60 s at a rate of 30 rolls per minute (1 s up, 1 s down), using a metronome (Figure 3).

#### 2.4.3. Vibration Rolling with Moderate Pressure Intensity (VRM)

For the VRM, the procedures were the same as the VRL, except the passive pressure pain intensity was maintained at 4–5 on a 10 mm VAS.

### 2.5. Statistical Analyses

All data analyses were performed using SPSS version 19 (Chicago, IL, USA) software. All results are reported as mean ± SD. The normal distribution of the data was checked by the Shapiro–Wilk test. Separate 2-way (time: pre-test vs. post-test) × 3 (intervention: GW vs. VRL vs. VRM) repeated measures ANOVAs were used to analyze the data. When appropriate, follow-up tests included one-way repeated measures ANOVAs with Bonferroni-adjusted pairwise comparisons, as well as paired samples *t*-tests. Cohen’s *d* values were reported to present the magnitude of the effect [24]. Significance for all the analysis was set a *p* < 0.05.

## 3. Results

At pre-test, there were no statistically significant differences among the three interventions for all dependent variables. 

### 3.1. Blood Pressure and Heart Rate

The results for systolic blood pressure, diastolic blood pressure, and heart rate are shown in Table 1. For the systolic blood pressure, the analysis showed no two-way time × intervention interaction (*p =* 0.98) and no main effect of intervention (*p* = 0.69), but there was a significant main effect for time (*p* = 0.002). After clasping across time, systolic blood pressure significantly increased from pre-test to post-test. For both diastolic blood pressure and heart rate, no two-way interaction, main effects for both intervention and time were found. 

### 3.2. Senior Fitness Test (SFT)

The results for senior fitness test outcomes are shown in Table 2.

#### 3.2.1. Back Scratch Test (BST)

A significant two-way interaction (time × intervention) was found for the BST (F = 23.69, *p* < 0.001). Subsequently, the effects of three interventions were investigated separately. Compared with the pre-test, the participants showed significant improvements in BST after VRL (*p* < 0.001) and VRM (*p* < 0.001), but not after GW (*p* = 0.79). In addition, there was a significant main effect of intervention (F = 3.97, *p* = 0.03), indicating greater BST after the VRL (*p* = 0.04) and the VRM (*p* = 0.04) than that after the GW.

#### 3.2.2. Chair Sit-and-Reach Test (CSRT)

For the CSRT, the time × intervention interaction (F = 1.16, *p* = 0.30) and main effect of intervention were not significant (F = 0.73, *p* = 0.41). However, the main effect of time was significant (F = 17.58, *p* = 0.001). With the time collapsed, the participants improved significantly in the CSRT after all three interventions (*p* < 0.001).

#### 3.2.3. 2 Min Step Test (2MST)

There was a significant two-way interaction (F = 13.41, *p* = 0.003). Compared to the pre-test, the participants showed significant improvements in 2MST after VRM (*p* < 0.001), but not after VRL (*p* = 0.95) and GW (*p* = 0.39). Additionally, participants showed significantly greater improvements after the VRM than those after the VRL (*p* = 0.03) and the GW (*p* = 0.02).

#### 3.2.4. 30 s Chair Stand Test (30SCST)

For the 30SCST, a significant two-way interaction was found (F = 29.87, *p* < 0.001). The follow-up tests indicated that participants showed significantly greater improvements after the VRL (*p* = 0.004) and the VRM (*p* = 0.002) than that after the GW.

#### 3.2.5. 30 s Arm Curl Test (30SACT)

There was a significant two-way interaction (F = 15.28, *p* = 0.002). The follow-up tests showed that the participants improved significantly in the 30SACT only after the VRM (*p* = 0.001). Additionally, participants showed significantly greater improvements after the VRL (*p* = 0.01) and the VRM (*p* = 0.003) than that after the GW.

#### 3.2.6. 8 Foot up and Go Test (8FUGT)

For the 8FUGT, a significant two-way interaction (time × intervention) was found (F = 18.87, *p* = 0.001). The follow-up tests showed that the participants improved significantly in the 8FUGT after the VRL (*p* = 0.001) and the VRM (*p* < 0.001), but not after the GW (*p* = 0.43). Additionally, participants showed significantly greater improvements after the VRL (*p* = 0.001) and the VRM (*p* = 0.03) than that after the GW.

## 4. Discussion

Regarding blood pressure, we discovered that post-test systolic blood pressure values after the GW, the VRL, and the VRM were significantly higher than pre-test. This discovery was inconsistent with the results of studies that have reported slightly reduced blood pressure after such interventions. Okamoto et al. recruited healthy young adults as participants and investigated the effect of body and lower limb fascia relaxation with rollers on peripheral vascular function. The results revealed that after the intervention, brachial–ankle pulse wave velocity significantly declined and plasma nitric oxide concentration significantly increased [6]. Ketelhut et al. had participants perform foam rolling on upper and lower body muscles, and found that after the intervention, systolic blood pressure, diastolic blood pressure, peripheral resistance, and arterial stiffness of the peripheral arteries significantly decreased [25]. A literature review by Cheatham et al. stated that the mechanism by which fascia relaxation with rollers reduces blood pressure may be related to the decline in activity in the sympathetic nervous system and the increase in the release of nitric oxide from the vascular endothelium [26]. Through a decrease in arterial stiffness and an increase in vasodilation, blood pressure can decrease. One difference between our study and those mentioned above is the study population, where the older individuals in the current study might have different cardiovascular responses than those in young healthy individuals. It is possible that the age-specific difference can be due to the increased vascular stiffness in older individuals [27,28], which the current warm-up protocols were not able to maintain to even decrease the blood pressure. Another possible reason why our results were inconsistent with those of the literature may be that the participants were expected to undergo physical fitness and balance function tests, where their sympathetic nervous system was in a more activated state, resulting in their blood pressure being slightly higher than during the pre-test. Regarding heart rate, no significant differences were observed between the pre-test and post-test, which was consistent with results of Ketelhut and Lastova et al. [25,29]. 

The overall testing performance from the SFT at baseline in the current study was better than those reported previously from different countries [23,30,31]. For the upper and lower limb flexibility, significant differences were observed between the pre-test and post-test for a low-intensity vibration roller intervention and a moderate-intensity VR intervention. The results revealed that vibration rollers immediately improved flexibility in healthy older women. This finding was similar to that obtained by Wilke et al. through meta-analysis [3], which was conducted with healthy young adults. Thus, VR was beneficial for both healthy young adults and older adults. The literature review performed by Marcucci et al. indicated that a decline in older adults’ joint ROM is associated with changes in their joints, muscles, tendons, and fasciae [18]. More specifically, joint changes include cartilage degeneration, joint capsule thickening, and ligament stiffness increases; muscle and tendon changes include muscle stiffness increases and tendon stiffness decreases; and fascia changes include stiffness increases [18]. Given that the effects of aging on joint ROM occur simultaneously in joints, muscles, tendons, and fasciae, we expected the effectiveness of VR in older adults to be less than that in young adults. We compared the results of the pre-test and post-test of back stretching and the chair sit and reach test, and discovered the trend that the effectiveness of a VMR was greater than that of a VML intervention. Phillips et al. compared the effects of rolling with different durations (60 and 200 s) on knee and ankle joint ROM, and the results indicated that longer duration had greater improvements than those of the shorter duration [32]. The results of these studies and of the present study imply that when the duration of rolling is longer and pressure is higher, the flexibility improvement is more satisfactory. Cheatham et al., however, compared the effectiveness of rollers with three levels of densities and observed that while all three rollers significantly increased joint ROM, the moderate density roller was more effective than the other two, though this was not statistically significant [33]. Thus, fascia relaxation with excessively high pressure or an excessively long duration could result in muscle flexibility decline caused by muscle contraction for protection and consequently result in injury.

Regarding upper and lower limb muscle strength, the post-test results for the VRM and the VML were significantly greater than that for GW, indicating that vibration rollers immediately improved muscle strength in healthy older women. The literature review conducted by Cheatham et al. indicated that the mechanism by which fascia relaxation with rollers improves muscle strength may be related to improvements in motor unit recruitment efficiency, muscle perfusion, and soft tissue stiffness reduction [26]. The meta-analysis performed by Wiewelhove et al. revealed that foam rolling has negligible effect on maximal strength and jump, and only a small improvement on sprint performance [34]. This indicates that although foam rolling does not improve muscle performance, it does not reduce muscle performance, either. We asked our participants to undergo a 30 s bicep arm flexion test and a 30 s chair stand test. For safety, the tests were lower in intensity, higher in repetition, and longer in duration than the tests conducted in other studies (i.e., maximal strength, jump, and sprint tests). The results demonstrated that VR may benefit more in medium-strength, high-repetition endurance activities. However, further verification is required.

Regarding cardiorespiratory endurance, we noticed that the post-test result for the VRM was significantly higher than those for the VRL and the GW, suggesting that vibration rolling with medium intensity can immediately improve cardiorespiratory endurance in older women. The results of the 2 min step test might have been affected by the oxygen supply efficiency of the peripheral blood vessels. As mentioned, fascia relaxation with foam rolling could increase the release of nitric oxide from the vascular endothelium and promote vasodilation [6,26], and peripheral vasodilation could increase the efficiency of oxygen supply to muscles during exercise, enabling participants to complete additional steps during the test. Notably, only the VRM intervention improved the step test results, implying that the effectiveness of VR on improving cardiorespiratory endurance is related to intensity.

We evaluated dynamic balance and agility with a timed up and go test and discovered significant differences between the pre-test and post-test results for the VRL and VRM interventions. The results also revealed significant differences when comparing VR interventions with the GW. This indicates that VR could immediately improve dynamic coordination and agility in healthy older women. Similarly, Lee et al. compared the effectiveness of warm-ups with VR, non-VR, and SS among healthy young adults and discovered that both vibration and nonvibrating rollers can significantly enhance flexibility, muscle strength, and dynamic balance [11]. In brief, the dynamic balance of both healthy young adults and older adults can be improved using rollers. We surmised that the observed improvements on the timed up and go test were related to increases in lower limb muscle strength. Given that the completion time of a timed up and go test is affected by numerous factors such as dynamic balance, muscle strength, coordination, and executive functions [35], we asked the participants to complete the test at the fastest safe speed rather than at their preferred speed, which could evidently highlight the effect of muscle strength on performance on the timed up and go test [36].

This study had several limitations. First and foremost, the current study may suffer from a relatively low sample size, given the large inter-subject variability among older adults. A larger sample with a broader age range (e.g., over 80 years of age) will be necessary for conducting future research. Second, we simultaneously examined the effectiveness of VR on cardiovascular parameters (i.e., blood pressure and heart rate) and mobility functions (i.e., flexibility, physical fitness, and dynamic balance). In this case, participants expected to undergo the movement tests, which could have affected their cardiovascular parameters. Third, because the participants of this study were older adults, who had difficulty operating the VR by themselves, the research staff had to implement the VR interventions. Thus, when comparing this study with other studies where self-administered rolling was performed, caution needs to be taken. Future study should explore the possible self-administered VR exercises that can be performed by older adults, and the potential benefits on activities of daily living. 

## 5. Conclusions

We conclude that vibration rolling is immediately effective on older women’s flexibility, muscle strength, cardiorespiratory endurance, and dynamic balance. The effectiveness of vibration rollers appears to be affected by the rolling pressure intensity. In practical situations, healthy older adults may adopt vibration rolling as a home exercise under proper supervision and guidance.

- Vibration rolling does not influence older women’s blood pressure parameters.
- Vibration rolling can improve acute flexibility, muscle strength, cardiorespiratory endurance, and dynamic balance.
- Under proper guidance, vibrating rolling can be used at home for older adults.

## Figures and Tables

**Table 1:** Mean ± Standard Deviation of systolic blood pressure, diastolic blood pressure, heart rate following three warm-up protocols.

	GW			VRL			VRM		
	Pre	Post	ES	Pre	Post	ES	Pre	Post	ES
SBP (mmHg)	132.08 ± 18.22	142.60 ± 19.49	0.56	130.87 ± 9.46	139.73 ± 20.26	0.56	130.67 ± 18.16	139.93 ± 21.85	0.46
DBP (mmHg)	76.73 ± 12.19	81.00 ± 13.20	0.34	77.20 ± 9.52	78.53 ± 12.46	0.12	75.87 ± 8.97	77.30 ± 11.86	0.14
HR (bpm)	72.27 ± 11.86	71.53 ± 13.24	0.06	73.80 ± 11.60	72.80 ± 10.48	0.09	75.00 ± 9.57	73.53 ± 10.76	0.14

GW: general warm up; VRL: vibration foam rolling with light pressure pain; VRM: vibration foam rolling with moderate pressure pain. SBP: systolic blood pressure; DBP: diastolic blood pressure; HR: heart rate.

**Table 2:** Mean ± Standard Deviation of shoulder (back scratch), lower-body flexibility (chair sit-and-reach), aerobic endurance (2 min step), lower-body strength (30 s chair stand), upper-body strength (30 s arm curl), agility and dynamic balance (8 foot up and go) before and after three warm-up protocols.

	GW			VRL			VRM		
	Pre	Post	ES	Pre	Post	ES	Pre	Post	ES
Back scratch (cm)	−1.30 ± 5.78	−1.38 ± 5.99	0.01	−1.10 ± 6.02	0.47 ± 5.23 *^#^	0.28	−1.40 ± 6.11	1.07 ± 5.43 *^#^	0.43
Chair sit-and-reach (cm)	5.89 ± 10.23	7.05 ± 11.19	0.11	5.91 ± 10.72	7.25± 11.51	0.12	5.71 ± 9.42	8.16 ± 11.51	0.23
2 min step (repetitions)	91.00 ± 29.23	90.27 ± 27.87	0.03	93.87 ± 29.92	94.07 ± 27.94	0.01	98.73 ± 22.02	108.20 ± 24.14 *^#+^	0.41
30s chair stand (repetitions)	19.66 ± 4.60	16.00 ± 2.56	0.98	19.33 ± 3.81	22.67 ± 6.34 ^#^	0.64	20.80 ± 5.86	25.13 ± 8.08 ^#^	0.61
30s arm curl (repetitions)	26.20 ± 5.19	24.93 ± 4.27	0.27	27.20 ± 5.02	29.00 ± 5.52 ^#^	0.34	24.86 ± 3.02	28.67 ± 4.22 *^#^	1.04
8 foot up and go (seconds)	6.80 ± 2.14	6.93 ± 1.94	0.06	6.57 ± 2.07	6.04 ± 1.90 *^#^	0.27	6.92 ± 2.24	6.25 ± 2.21 *^#^	0.30

GW: General warm up; VRL: vibration foam rolling with light pressure pain; VRM: vibration foam rolling with moderate pressure pain. *: Significant difference compared with pre-test (*p* < 0.05) ^#^: Significant difference compared with GW (*p* < 0.05) ^+^: Significant difference compared with VRL warm up (*p* < 0.05).

## Data Availability

The data are available upon request to corresponding author’s email.
